# Hearing Loss in Patients with Diabetes Mellitus

**DOI:** 10.1016/S1808-8694(15)30498-5

**Published:** 2015-10-19

**Authors:** Thiago Hernandes Diniz, Heraldo Lorena Guida

**Affiliations:** 1 Speech and Hearing Therapist. Researcher on Speech and Hearing Therapy; 2 Assistant Professor of the Speech and Hearing Therapy Department - UNESP - Marìlia Campus -SP. UNESP - Philosophy and Science School - Marìlia Campus -SP

**Keywords:** audiology, diabetes mellitus, hearing loss

## Abstract

The relationship between diabetes mellitus and hearing loss is described as ‘controversial’ in the literature, given that in the clinical realm many patients present dysacusis while others do not.

**Aim:**

this study aims to investigate the relationship between hearing loss and diabetes mellitus and add to the knowledge being developed in this area.

**Study design:**

cross-sectional clinical trial.

**Materials and method:**

in our study we analyzed the medical charts of 50 adult patients of both genders, aged above 45 years, and diagnosed with diabetes mellitus, seen in our institution in 2006 and 2007, and compared them to the charts of 50 metabolic disease-free individuals.

**Results:**

this study found statistically significant worse audiometric thresholds among patients with diabetes mellitus when compared to patients in the control group.

**Conclusion:**

the results identified in this study point to a correlation between hearing loss and diabetes mellitus. This possibility should therefore be further investigated by health care workers providing care to patients with diabetes mellitus, in the form of closer follow-up on the auditory health of this patient group.

## INTRODUCTION

Diabetes mellitus (DM) is a genetically inherited disease in which glucose serum levels are abnormally high due to relative or absolute insulin deficiency. Its clinical manifestation is characterized by metabolic disorders, and vascular and neuropathic complications. DM is yet an incurable disease and its management should be focused on preventing chronic complications^1^.

Diffuse thickening of basal membranes, also observed in the vascular endothelium, is one of the most prevalent morphologic findings in DM, referred to as diabetic microangiopathy. Such thickening is more evident on skin capillaries of skeletal muscles, retina, kidney glomeruli, and renal medulla. The pathogenesis of this morphologic disorder is yet unclear, but it is directly related to hyperglycemia. There also are morphologic disorders related to the involvement of lower extremity motor and sensorial nerves, characterized by injuries to Schwann cells, myelinic degeneration, and axonal damage. The cause of this neuropathy is still controversial, but it may be related to diffuse microangiopathy and the consequent malnourishment of peripheral nerves. Arteriosclerosis, frequently seen in conjunction with DM, may also contribute to the onset of neuropathies, as it interferes with the nutrient transfer rate^2^, ^3^.

Angiopathy may occur both directly - interfering with cochlear blood supply and reducing nutrient transportation as a result of capillary wall thickening - and indirectly - reducing flow in a narrowed vasculature or, yet, causing secondary degeneration of the eighth cranial nerve^4^.

Studies carried out on rats found thickened walls in the vessels of the modiolus, increased basal membrane thickness in the stria vascularis capillaries and increased loss of outer hair cells (this last feature was found in diabetic rats exposed to noise). These studies were disputed, as drugs were given to the rats to induce DM, thus not reflecting the actual physiological and genetic mechanism that leads to the onset of DM in human beings^5^, ^6^, ^7^, ^8^.

Pathology tests carried out in temporal bones of diabetic individuals of various age ranges have shown that the changes in stria vascularis capillary wall thickness - they were 10 to 20 times thicker than usual - were similar to those found in atherosclerosis, although they were more pronounced and limited to the stria vascularis. The authors have also observed other forms of degeneration, but they were also seen in temporal bones of non-diabetic individuals of the same age ranges. They also reported that these alterations are typically found in DM patients, but that they are not DM-specific, observing that atherosclerosis is reduced peripherally, whereas diabetic angiopathy increases in intensity in small vessels^9^.

Another study performed on temporal bones detected thickened stria vascularis and modiolus capillary thickening^5^.

Capillary wall thickening and the consequently reduced vessel lumen will more intensely affect the internal auditory artery^3^.

Other pathology test-based studies done in humans described spiral ganglion neuron atrophy and eighth cranial nerve demyelination in diabetic individuals, indicating that demyelination is also an early injury to extremity peripheral nerves in DM and that myelin metabolic disorders may be relevant in the pathogenesis of diabetic neuropathy. The following findings were observed under optic microscopy: auditory nerve demyelination by degeneration of the myelin sheath, with minor axonal alterations and perineurium fibrosis; severe spiral ganglion atrophy with cell loss in the cochlear basal and middle turns, accompanied by decreased number of nerve fibers in the spiral lamina. Other findings are: reduced number of ganglion cells in the ventral and dorsal cochlear nuclei, small loss of ganglion cells in the superior olivary complex, inferior colliculus and medial geniculate body. No DM-specific alteration was found in the auditory centers of either temporal lobes^3^.

Some authors believe that angiopathy is the primary injury connected to hearing loss, while others credit such role to neuropathy, arguing that vessel wall thickening is quite unspecific and also found in other diseases. Human audiology studies indicate that the thresholds of patients with peripheral neuropathy were always worse than control group individuals in any frequency^10^.

Hearing loss may also have its origins in congenital or acquired genetic mutations affecting nuclear and mitochondrial genes in both syndromic and non-syndromic manifestations8. Mitochondrial DNA mutations are transmitted through maternal genes, but spontaneous mutations may also occur. Mitochondrial disorders may lead to cell death^11^, ^12^.

In a study done on a particular genogram in which nine children of mothers with DM also developed the disease, coinciding hearing loss of varied severity was observed in each diabetic individual. The authors also observed that in the third generation of this family diabetes and hearing loss were present only in the offspring of affected mothers. Such finding strongly suggested that inheritance is exclusively maternal, a trait found in diseases associated with mitochondrial DNA mutation^13^.

Other studies reported that DM may be part of a genetically inherited syndrome in which diabetes mellitus is merely one of the syndrome's symptoms: Wolfram syndrome, also called DIDMOAD (Diabetes Insipidus, Diabetes Mellitus, Optic Atrophy and Deafness)^14^.

Some authors have mentioned DM as a possible cause for sudden hearing loss. In spite of other causing factors such as atherosclerosis and viral infection, DM is seen as the main cause of deafness due to diabetic microangiopathy^15^.

Many papers have tried to identify the relationship between hearing loss and DM. Such studies are justified as controversy still looms this topic, and given that while many diabetic patients develop hearing loss, many do not. Another fact is that most of the time DM will involve older patients, thus making it even harder to connect hearing loss to DM due to presbycusis.

A study compared 51 diabetic patients aged between 8 and 21 years against a control group of 13 patients and found no statistically significant differences between their audiological test results^16^.

However, contradictory results were found in other studies on the same topic. Another study looked at 30 diabetic patients and compared them to 30 healthy individuals, all of which aged 50 or more, only to find higher prevalence rates of high frequency hearing loss among diabetic patients. No connections were however established between hearing loss and disease duration^17^. There is also a study that correlates hearing loss and disease duration, without however setting clear criteria to pick participants of the control group, thus questioning the validity of its findings^18^.

Other studies suggest that there could be injuries in various points of the auditory pathways, possibly due to neural defects or cochlear injury. Results indicate that the hearing loss patterns found in DM patients do not comply with what is observed in senility-related hearing loss in terms of frequency distribution^10^.

Another study found high-frequency sensorineural hearing loss in 37.5% in the patient population. However, these results were not compared against a control group^19^.

This paper aims to describe the audiological characteristics of diabetes mellitus patients and compare these findings against to the test results of DM-free individuals of similar age groups.

## MATERIALS AND METHOD

This study was carried out in the Clinical Audiology Division of a health care center located in the countryside of the State of São Paulo, Brazil. The study was approved by the Research Ethics Committee of our institution under permit 1494/2006.

The medical charts of 50 diabetes mellitus patients of both genders (66% females and 34% males) aged between 45 and 83 years (mean 67.56 years) sent for audiological examination in the years of 2006 and 2007 were examined^17^.

We collected information on the patients' audiological history, identification data and hearing health history; tone threshold audiometry, to assess tone thresholds (air and bone conduction)^20^; logoaudiometry, to evaluate speech recognition index (SRI) or speech reception threshold (SRT)^21^; impedance test, to analyze tympanometry and acoustic reflex data of the stapedius muscle (these procedures looked at the functional integrity of the ossiculartympanic complex)^22^.

Audiometry and logoaudiometry tests were performed in a soundproof booth, with audiometer GSI 61 Grason - Stadler. Acoustic impedance test measurements were performed with a GSI 38 Grason - Stadler device.

The data sets were analyzed in terms of audiometric findings: hearing loss level^23^, ^24^. Logoaudiometry provided the data required to confirm the audiometry findings to assist in topographic diagnosis^21^. ANOVA^25^ was used to verify statistically significant differences between the mean hearing threshold values.

Tympanometry results were classified as proposed by Jerger^26^ and acoustic reflex was analyzed as present or absent.

## RESULTS

The main results obtained from audiological research in the group of DM patients were the following: dysacusis (74%), high blood pressure (72%), tinnitus (70%), recruitment (46%), aural fullness (44%), otalgia (36%), dizziness (32%), autophony (30%), vertigo (30%), pruritus (24%), and oozing (4%). The main findings gathered from the control group were: dysacusis (50%), tinnitus (46%), recruitment (30%), aural fullness (24%), vertigo (22%), autophony (14%), otalgia (14%), dizziness (14%), and oozing (4%).

No statistically significant differences were found between the age mean values and standard deviations for the case (65.50±8.48) and control 67.56±9.55) groups (Anova p=0.256 / a=0.05).

In the case group, acoustic reflex was present in 72% and absent in 28% of the patients. Acoustic reflex was present in 41% and absent in 59% of the control group subjects.

Tone audiometry tests identified the following types of hearing loss among case group members: sensorineural (38%), mixed (24%), and conductive (1%); 37% of the audiograms in this group were considered as normal. Control group members had the following types of hearing loss: sensorineural (26%), mixed (4%), and conductive (2%); 68% of the audiograms in this group were deemed normal.

In order to facilitate analysis, the results for impedance tests and hearing loss levels are presented in the form of tables considering each analyzed variable.

Table 1 presents the data on impedance tests performed on DM patients compared against the data gathered from control group individuals (100 ears for each group).

**Table 1:** Impedance test results for the control group (CG) compared against the diabetes mellitus group (DM); analysis of 100 ears from each group.

	CG	DM
A	88%	80%
B	2%	10%
Ad	6%	6%
As	4%	4%

Table 2 compares the audiometry test results considering the differences in mean values between the case and control groups.

**Table 2:** Statistical analysis results; audiometric thresholds (dB) mean values and standard deviation according to Davis & Silvermann24 (DS) and BIAP25 for the two groups - control group (CG) vs. diabetes mellitus group (DM).

	CG	DM	Anova p value
BIAP RE	24.82±12.28	38.25±21.36	0.000208^a^
BIAP LE	24.17±12.67	39.40±20.96	0.000028^a^
DS RE	21.88±11.68	35.18±20.55	0.000133^a^
DS LE	20.76±12.44	35.80±19.53	0.000013^a^

a significant for a = 0.001

Table 3 shows data on the comparison between the two types of classification schemes used in this study (Davis & Silvermann^23^ and Recommendation 02/1 from the Bureau International d'Audio Phonologie - BIAP^24^) within each of the groups.

**Table 3:** Statistical analysis results; audiometric thresholds (dB) mean values and standard deviation, Davis & Silvermann24 (DS) vs. BIAP25, for the two groups - control group (CG) vs. diabetes mellitus group (DM).

	BIAP	DS	Anova p value
GC RE	24.82±12.28	21.88±11.68	0.222^a^
GC LE	24.17±12.67	20.76±12.44	0.176^a^
DM RE	38.25±21.36	35.18±20.55	0.465^a^
DM LE	39.40±20.96	35.80±19.53	0.376^a^

a non-significant for a = 0.05

Table 4, Table 5 contain the analysis on the level of hearing loss for the case group, while Table 6, Table 7 contain the same data however for the control group.

**Table 4:** Audiometric test results for right and left ears; patients in the diabetes mellitus group, according to hearing loss level - Davis & Silvermann^24^.

Level	Right ears		Left Ears		Total	
	n	%	n	%	n	%
Normal	20	40	16	32	36	36
Mild	8	16	18	36	26	26
Moderate	21	42	15	30	36	36
Severe	1	2	1	2	2	2

**Table 5:** Audiometric test results for right and left ears; patients in the diabetes mellitus group, according to hearing loss level - BIAP^25^

Level	Right ears		Left Ears		Total	
	n	%	n	%	n	%
Normal	14	28	12	24	26	26
Mild	12	24	12	24	24	24
Moderate grade 1	11	22	15	30	26	26
Moderate grade 2	11	22	8	16	19	19
Severe grade 1	2	4	2	4	4	4
Severe grade 2	–	–	1	2	1	1

**Table 6:** Audiometric test results for right and left ears; patients in the control group (DM-free subjects) according to hearing loss level - Davis & Silvermann^24^.

Level	Right ears		Left Ears		Total	
	N	%	n	%	n	%
Normal	36	72	34	68	70	70
Mild	11	22	12	24	23	23
Moderate	6	6	4	8	7	7

**Table 7:** Audiometric test results for right and left ears; patients in the control group (DM-free subjects) according to hearing loss level - BIAP^25^.

Level	Right ears		Left Ears		Total	
	N	%	n	%	n	%
Normal	21	42	24	48	45	45
Mild	25	50	20	40	45	45
Moderate grade 1	4	8	6	12	10	10

## DISCUSSION

Tone audiometry test results were significantly worse among diabetic patients when compared to control group individuals from the statistical standpoint, thus eliciting a correlation between DM and hearing loss in our group of subjects^27^. Audiometry test results were also statistically compared after being categorized by two different methods^3^, ^24^, but no statistically significant difference was found between the two quantitative classification approaches. Qualitatively, however, there was an increase on the number of subjects with normal mean speech frequency when Davis & Silvermann^23^ was used in comparison to BIAP^24^.

According to Jerger^26^, tympanometric A-curves are found in individuals with normal middle ears, while As-curves refer to stiff tympanic-ossicular systems, and Ad-curves show tympanic-ossicular system laxity. B-curves are present in individuals with fluid in their middle ears, and C-curves are related to Eustachian tube disorders.

A-curves were found in 80% of the DM group members and in 88% of the control group subjects, as they can be related to normal hearing and sensorineural hearing loss. This finding matches the main type of hearing loss diagnosed in our study (sensorineural). The number of B, Ad, and As-curves in the DM group was higher when compared to the control group, as more mixed and conductive hearing loss cases were also seen in the case group.

Acoustic reflex is an involuntary contraction of the stapedius muscle in response to sound stimuli. Normal threshold values range between 70 and 100 dB, and are situated usually 85 dB above the hearing threshold^21^. This was found in 28% of the DM patients and in 41% of the subjects in the control group. Apart from hearing losses, conductive components also account for the differences found between the groups, as a greater number of mixed and conductive were found in the DM group^21^.

The widespread presence of high blood pressure in this population points to angiopathy as one of the main etiologies, as described by Robbins^2^, Makishima^3^and Taylor^4^.

No statistically significant differences were found in the mean ages of the DM and control group members, as described by Frisina^28^ and Sasso^29^.

The data gathered from the audiological interview indicated that the main complaint from DM group members was dysacusis, followed by high blood pressure, and tinnitus. Fukui^15^ identified diabetes, hearing loss, high blood pressure, and tinnitus as the main clinical factors manifested by DM patients. Such finding matched audiometry test results, as they revealed a predominance of bilateral sensorineural hearing loss^30^. Such prevalence of bilateral sensorineural hearing loss may be related to hyperglycemia, i.e., a systemic disease that results in bilateral disorders. Tympanometric curves and acoustic reflex findings were compatible with tone audiometry results.

A paper published recently showed similar findings, indicating that DM is associated with hearing loss in middle-aged individuals. This paper is the only that seems to present such association at a larger scale^27^.

## CONCLUSION

The findings and results gathered in this study point to the existence of a relationship between hearing loss and diabetes mellitus. Therefore, the auditory health of DM patients is to be more carefully and thoroughly followed up by health care workers dealing with this condition.

## ACKNOWLEDGEMENT

To the São Paulo State Research Support Foundation (FAPESP - Process # 2006/04810-3) for the grant given to this research project.
